# Predictors of affect across social contexts: An integration of existing diary datasets

**DOI:** 10.1111/bjso.70124

**Published:** 2026-08-14

**Authors:** Anna Tovmasyan, Denethri Gamagedara, Kou Murayama, Julie Bowker, Robert Coplan, Jennifer Lay, Thuy‐vy Nguyen, Dongning Ren, Olga Stavrova, Virginia Thomas, Dwight Tse, Netta Weinstein

**Affiliations:** ^1^ School of Psychology and Clinical Sciences University of Reading Reading UK; ^2^ School of Psychology and Clinical Language Sciences University of Reading Reading UK; ^3^ Hector Research Institute of Education Sciences and Psychology University of Tübingen Tübingen Germany; ^4^ Department of Psychology University at Buffalo Buffalo New York USA; ^5^ Department of Psychology Carleton University Ottawa Ontario Canada; ^6^ Department of Psychology University of Exeter Exeter UK; ^7^ Department of Psychology Durham University Durham UK; ^8^ Department of Work and Social Psychology Maastricht University Maastricht the Netherlands; ^9^ Department of Social Psychology and Microsociology University of Mannheim Mannheim Germany; ^10^ Department of Social Psychology University of Tilburg Tilburg the Netherlands; ^11^ Department of Psychology Middlebury College Middlebury Vermont USA; ^12^ Department of Psychological Sciences and Health University of Strathclyde Glasgow UK; ^13^ Oxford Internet Institute University of Oxford Oxford UK

**Keywords:** affect, age, extraversion, social interactions, solitude

## Abstract

Daily life is characterized by periods of complete and public solitude, face‐to‐face interactions and digitally mediated interactions when alone or with others, all of which elicit varying degrees and different types of affect. This study aimed to provide a comprehensive understanding of affect during these distinct everyday contexts. By combining data from 11 distinct datasets, we examined how time spent in complete solitude (not interacting and with no‐one around), digitally mediated interactions when alone, public solitude (not interacting but with others present), interacting face‐to‐face and digitally mediated interactions when with others, as well as individual differences in age and extraversion, were associated with four types of affect distinguished on two dimensions (arousal and valence) and loneliness. Results showed that prior face‐to‐face interactions were associated with increased high‐arousal positive affect and decreased low‐arousal positive affect. This research offers nuanced insights into how social and solitary contexts, personality and age contribute to everyday affective experiences.

## INTRODUCTION

Our well‐being is formed through the experiences incurred across a variety of life's everyday settings, which vary in their social qualities and include complete solitude (i.e. being physically alone and not interacting), digitally mediated interactions when alone, public solitude (physically with others around but not interacting; Weinstein et al., 2023), face‐to‐face interactions and digitally mediated interactions when with others. Although it is recognized that both the quantity and quality of those contexts may together shape affect—a range of emotional experiences that characterize our days, little integrated work has been conducted to identify how these key contexts contribute, in balance, to positive and negative affects indicative of well‐being and whether psychological and demographic factors may impact how individuals feel within those contexts and how the contexts overall contribute to well‐being. This is in part because much of the existing literature has devalued the role of complete and public forms of solitude as a potential contributor to daily well‐being and focused solely on social interactions (Weinstein et al., 2021, 2022, 2023). The present research aimed to address this gap in our understanding by synthesizing existing daily diary studies focused on understanding both solitude and social interactions and examining their unique and shared contributions to positive and negative affect.

### Contexts within everyday life

While extensive research has shown that face‐to‐face social interactions can be associated with reduced loneliness, increased positive affect and reduced negative affect (Pauly et al., 2018; Sun et al., 2020; Tovmasyan et al., 2023), findings have been mixed across life's other contexts. For example, solitude has been viewed as a double‐edged experience, sometimes evoking feelings of peace and relaxation, yet at other times linking to loneliness and emotional distress (Coplan et al., 2019; Lay et al., 2019). Its potential to produce both positive and negative affect may be due, in part, to the fact that solitude is able to fulfil certain functions and needs of individuals (e.g. self‐connection, efficacy, choiceful action; Weinstein et al., 2021), while failing to meet other social needs, and social connection in particular (e.g. relatedness to others; see Self‐Determination Theory, Ryan & Deci, 2000; Social Baseline Theory, Beckes & Coan, 2011). Across studies, spending time in solitude—in both public and complete forms (i.e. with others around or not around)—has been linked to increased positive affect (Coplan et al., 2019; Weinstein et al., 2023), negative affect (Killeen, 1998) and loneliness (Tsang et al., 2022), both in its own right, and when compared to social interactions. Other findings identify no affective costs or benefits to public forms of solitude (Chui et al., 2014). A similar lack of clarity has surrounded findings regarding affect following digitally mediated interactions; some studies suggest that digital contact reduces loneliness and enhances positive affect (Sahi et al., 2021; Seabrook et al., 2016), while others report increased negative affect following digital interaction (Keles et al., 2020) or no significant associations with affective outcomes, such as depression, happiness and loneliness (Tsang et al., 2022).

### Considerations when comparing everyday life contexts

Taken together, the literature reviewed above suggests that whether people spend their day in the company of others, on their own or interacting digitally contributes to positive and negative affect, but mixed findings leave open the question of how each context contributes to more positive or more negative affect within and across a day. The question is further complicated because positive and negative distinctions between affects overlook another important dimension that characterizes affect, namely, arousal level—a quality that might fundamentally influence the relationship between social and solitudinal contexts and affect. The Circumplex Model of Affect (Russell, 1980) describes a nuanced way of understanding affect, mapped along two dimensions: valence (positive to negative) and arousal (high to low). However, while the Circumplex Model allows researchers to look at affective states more holistically, there is evidence to suggest that positive affect and negative affect are relatively independent (though related) dimensions: they are characterized by different patterns of neural activity (Cacioppo et al., 1997; Carver & Scheier, 1990; Kim & Hamann, 2007), by divergent appraisal styles (Diener & Emmons, 1984; Smith & Lazarus, 1993), and by distinct personality traits (Gross & John, 2003; Larsen & Ketelaar, 1991). Furthermore, the presence of positive affect does not necessitate the absence of negative affect, nor does the absence of one state dictate the presence of another (Larsen et al., 2001; Larsen & McGraw, 2014). Therefore, the current study opted to consider affect as four distinct entities: high‐arousal positive, high‐arousal negative, low‐arousal positive and low‐arousal negative.

The quality of specific affective experiences on both valence and arousal dimensions may be key to understanding how affect forms in everyday social and solitude contexts. For example, solitary time can provide a unique opportunity to rest and rejuvenate (Nguyen et al., 2019; Weinstein et al., 2021). This phenomenon, termed the deactivation effect (Nguyen et al., 2018), is frequently reported by participants of qualitative studies as one of the most important characteristics of solitude (Ost Mor et al., 2021) that results from the stillness and lack of external stimulation often associated with solitude (Weinstein et al., 2021). If low‐arousal positive affect were conflated with high‐arousal positive affect, the affective benefit of solitude would therefore be diminished or eliminated altogether. Similarly, people may feel more high‐arousal (e.g. excitement and anger) and less low‐arousal (e.g. calm and bored) emotions when interacting with others (e.g. Kanning et al., 2021). If arousal is ignored, these contrasting effects may cancel each other out. Therefore, when examining associations between solitudinal or social contexts and affect, it is important to account for affective arousal rather than focusing solely on affective valence.

Comparisons of affect across the solitudinal, social and digitally mediated contexts may be further affected by individual differences that influence how people approach and experience solitude. Research has increasingly pointed to the utility of the introversion‐extraversion dimension for understanding variations in affect across life's contexts. Introverts gain energy from time alone (e.g. Helgoe, 2013), while extraversion on the opposite end of the continuum is characterized by sociability, assertiveness and high activity (McCrae & Costa, 1987). Those higher in introversion are generally believed to prefer and benefit from solitude and to find virtual and face‐to‐face interactions anxiety‐provoking (Rice & Markey, 2009).

Introverts should consistently derive more positive affect from solitude, but in studies to date, the associations between contexts and introversion remain inconclusive (Nguyen et al., 2022). For example, Long et al. (2003) found that introverts feel lonelier in solitude, suggesting that introverts might not always find solitude beneficial, perhaps because their anxieties interfere with their time alone. Counterintuitively, studies have shown that those higher in extraversion tend to choose solitude more often (Thomas & Nelson, 2024), and value certain solitary activities such as exploring nature (Ren et al., 2025).

A second key focus in the literature on social and solitude context costs and benefits concerns developmental differences. A body of work shows older adults experience fewer affective ‘costs’ of solitude relative to younger adults (Lay et al., 2018, 2019). As individuals age, they build constructive intentions around their solitude time and feel more choiceful in these experiences (Coplan et al., 2019, 2021), leading to peaceful affect and lower loneliness during solitude (Ost Mor et al., 2021; Weinstein et al., 2021). Notably, these benefits are not specific to solitude. Older adults also tend to experience a boost in well‐being following digital (Tsang et al., 2022) and face‐to‐face social interactions (Luo et al., 2022).

Affect and social context could be studied either in the lab or naturalistically; however, if the focus of the question is on changes in *everyday* affect, the second approach is more pertinent. Naturalistic approaches, such as diary or experience sampling methods, provide ecological validity and allow researchers to minimize retrospective bias (Shiffman et al., 2008). Here, participants are instructed to record, in structured ways, feelings that occurred during the day. However, such studies occur in uncontrolled environments, and it is therefore possible that findings may be impacted by extraneous factors that are not captured by the research methods used in these studies (Monk et al., 2015). Nevertheless, increasing sample size may help tackle this issue. Yet, the mean sample size in the average EMA‐type study is 137 (Wrzus & Neubauer, 2023), which may not be sufficient to control for changing environmental factors. The approach we take in the current study—integrating daily diary datasets—helps with increasing power and therefore helps minimize the issues that come up when having an uncontrolled environment.

### Current project

The current study integrates data from 11 diary‐format datasets to examine, through well‐powered analyses, how affect varies across different social contexts: complete solitude, digitally mediated interactions when alone, public solitude and interacting face‐to‐face and digitally mediated interactions when with others. Drawing on the Circumplex Model of Affect (Russell, 1980), we examine how these contexts relate to both affective valence (positive vs. negative) and arousal (high vs. low), and we separately model loneliness as a key affect‐related outcome in this literature (Coplan et al., 2021). Further, we investigate whether individual differences in extraversion and age moderate these relationships, addressing existing gaps in the literature and offering a more nuanced understanding of how social context shapes emotional experiences. We address the following research aims:

Aim 1: Examine the experience of affect across different solitudinal and social contexts (complete solitude, public solitude, face‐to‐face, digital, a mix of face‐to‐face and digital).
Aim 2: Examine whether extraversion moderates the relationship between affect and solitudinal or social contexts.
Aim 3: Examine whether age moderates the relationship between affect and solitudinal and social contexts.
Aim 4: Examine how the amount of time spent in different solitudinal or social contexts is associated with changes in affect.

## METHOD

### Transparency and openness

The project was initially created to examine factors associated with affect in solitude. This paper is the result of a secondary exploratory analysis of the data, without any a priori hypotheses. The methods and results of the original project, as well as datasets and analyses scripts for the presented project, can be found on the Open Science Framework (OSF): https://osf.io/6deg4/?view_only=fe5bd431e6264d3d89f7b2cee971634a.

### Operational definitions

To date, there is no consensus in the literature on what constitutes solitude. In the current paper, we defined complete solitude as a state of being physically alone and not interacting with anyone digitally or face‐to‐face (Lay et al., 2020). Nevertheless, we acknowledge that certain studies included in our analysis may not conform to this definition. We extracted data that adhered as closely as possible to this definition, specifically with respect to whether digitally mediated interactions were accounted for. Where possible, we prioritized studies that explicitly accounted for the presence or absence of digitally mediated interactions; however, studies that did not address this distinction were also included where relevant.

In relation to affect, the most commonly studied social/solitary contexts in the literature are complete solitude, digital interactions and face‐to‐face interactions. However, having examined the data, we observed that they were mainly collected across five distinct contexts: complete solitude (not interacting and with no‐one around), digitally mediated interactions when alone (interacting with others digitally with no‐one around), public solitude (not interacting but with others present), face‐to‐face interactions (interacting with others who are physically present) and digitally mediated interactions when with others (interacting with others digitally with other people physically present). After examining the differences and similarities between the datasets, the decision was made to run two sets of models for each outcome: first, examining how time spent in each social context is associated with affect and, second, examining differences in affect at the time of being in each context.

### Data collection procedures

We pre‐registered the following: ‘all researchers who we can find who have published on solitude/alone since 2012 will be contacted’. In line with this, the search was conducted in Google Scholar using the terms ‘solitude’ OR ‘alone’ to identify active labs conducting research on affect in solitude between 2012 and 2022. As the aim was researcher identification rather than literature retrieval, labs rather than individual records were screened, with nine active labs identified. The search was supplemented by the last author's knowledge of the field. Labs that focused on studying loneliness rather than solitude as a state were excluded. While our pre‐registration also states that ‘we will contact all researchers with recent (last decade; 2012 on) and relevant output in the solitude literature’, it is important to acknowledge that the lab identification process was not systematic and relevant labs may therefore have been missed; however, no labs known to the authors were excluded.

Because the focus of the study was everyday solitude, only authors who used diary methods (i.e. studies that ask participants to report on their experiences at the end of each day) or experience sampling method (ESM, i.e. studies that prompted participants multiple times a day to report on their experience since the last assessment) were contacted. Of the nine labs identified, seven were contacted for data sharing by the lead author, the remaining two being the lead author's own lab and previous lab. All contacted researchers responded; however, one did not have usable data for the purposes of the analysis and was therefore not included, leaving a sample of six active researchers. Four authors provided one dataset each, one author provided three, one author provided two, and two datasets were provided by the lead author from the previous lab. Our own lab did not have a usable dataset due to the format of data collection, giving a final total of 11 datasets, which were collated on an ongoing basis. Table 1 summarizes the details of the studies used in the analyses; full eligibility criteria are available on the OSF.

**TABLE 1:** Summary of the included studies.

Study	Author	Year of data collection	Study type	Compliance rate (%)	*N*	Age	Gender	Affect measure
1	Tse	2020	Diary, 21 days	99.88	195	Young adults: *M* = 22.74 (*SD* = 4.21); Older adults: *M* = 64.36 (*SD* = 4.41); Range = 18–66	Young adults: 39% male, 61% female; Older adults: 37% male, 63% female	Nine items (0–4 scale), HAPA (*α* = .88); LAPA (*α* = .68); HANA (*α* = .81); LANA (*α* = .82)
2	Thomas	2020	Diary, 7 days	92.6	69	Range 18–35	57% female, 42% male, 1% unknown	Seven pairs of items (5‐point scale); HAPA (*α* = .71), LAPA (*α* = .51), LANA (*α* = .83), HANA single‐item (bored)
3	Nguyen	2021	Diary, 27 days	52.9	82	*M* = 19.36 (*SD* = 0.98), Range = 18–23	15% male, 83% female, 1% other	32 items (binary); HAPA (*α* = .93), LAPA (*α* = .90), HANA (*α* = .85), LANA (*α* = .84)
4	Nguyen	2021	ESM, 7 days	55.6	54	*M* = 20.70 (*SD* = 1.31), Range = 18–23	78% male, 22% female, <1% other	32 items (binary); HAPA (*α* = .86), LAPA (*α* = .85), HANA (*α* = .76), LANA (*α* = .78)
5	Lay	2021	Diary, 14 days	90.8	104	*M* = 19.88 (*SD* = 3.76), Range = 18–45	17% male, 82% female, 1% non‐binary	Nine items (0–100 scale); HAPA (*α* = .68), LAPA (*α* = .73), HANA (*α* = .66), LANA (*α* = .55); loneliness
6	Lay	2022	Diary, 14 days	55	45	*M* = 20.71 (*SD* = 4.02), Range = 18–42	16% male, 84% female	Seven items (0–100 scale); HAPA (*α* = .65), LAPA (*α* = .71), HANA (*α* = .83), LANA (sad only); loneliness
7	Lay	2019	Diary, 7 days, 5x/day	97.8	416	*M* = 19.45 (*SD* = 1.50), Range = 18–34	50% male, 50% female	16 items (1–3 scale); HAPA (*α* = .85), LAPA (*α* = .70), HANA (*α* = .88), LANA (*α* = .76)
8	Bowker	2022	ESM, 7 days, 5×/day	93	66	*M* = 20.38 (*SD* = 4.29), Range = 18–42	9% male, 86% female, 3% non‐binary	13 items (0–100 scale); HAPA (*α* = .65), LAPA (*α* = .90), HANA (*α* = .93), LANA (tired, loneliness)
9	Ren	2020	ESM, 7 days, 5×/day	83.3	265	Not reported	Not reported	Four items (1–5 scale): HAPA (happy), HANA (angry), LANA (sad, bored; *r* = .23).
10	Tovmasyan	2020	ESM, 7 days, 3×/day	86	103	Not reported	Not reported	PANAS; HAPA (*α* = .91), HANA (*α* = .86).
11	Tovmasyan	2021	ESM, 7 days, 3×/day	42	87	Not reported	Not reported	PANAS; HAPA (*α* = .92), HANA (*α* = .92).

### Outcome variables

The following affect measures were considered as outcomes: *loneliness* (assessed via asking participants to rate their level of loneliness when in different contexts), *High‐Arousal Positive Affect* (assessed via averaging all High‐Arousal Positive Affect items in the dataset, e.g. happy, cheerful, excited), *High‐Arousal Negative Affect* (assessed via averaging all High‐Arousal Negative Affect items in the dataset, e.g. angry, frustrated, nervous), *Low‐Arousal Positive Affect* (assessed via averaging all Low‐Arousal Positive Affect items in the dataset, e.g. calm, relaxed, at ease) and *Low‐Arousal Negative Affect* (assessed via averaging all Low‐Arousal Negative Affect items in the dataset, e.g. bored, sad, tired). See Table 1 for the measures used and reliability scores for each study.

Affect was assessed using a variety of scales across studies, including unipolar ratings (e.g. 0–4 Likert, 0–100 visual analogue, binary adjectives, PANAS) and one study that used bipolar adjective pairs (e.g. Happy‐Sad, Anxious‐Calm). To ensure conceptual consistency across datasets, bipolar items were reverse‐coded where necessary so that higher scores always indicated greater intensity of the target affect (e.g. happiness for High‐Arousal Positive Affect, sadness for Low‐Arousal Negative Affect). The scores for each measure were *z*‐converted for each dataset to ensure consistency across measures. This procedure placed each participant's scores on a common metric reflecting relative deviation from their study's mean, enabling comparison of associations rather than absolute levels across studies.

### Predictor variables

#### Age

Participants' age at baseline.

#### Extraversion

Studies assessed extraversion by adapting three or eight items from the Big 5 inventory (John et al., 1991) or The International Personality Item Pool (IPIP; Goldberg et al., 2006). The average score across items of a measure was *z*‐converted for each dataset to ensure consistency across datasets.

#### Weekday

Day of the week on which participants completed the measurement.

#### Depression

Depression was assessed using PHQ‐4 (items one and two; Kroenke et al., 2009), PHQ‐9 (Kroenke et al., 2001), BDI‐21 (Beck et al., 1996), DASS‐21 depression subscale (Henry & Crawford, 2005) and CES‐D (Radloff, 1977). The average score across items of a measure was *z*‐converted for each dataset to ensure consistency across datasets.

#### Anxiety

Studies assessed anxiety using PHQ‐4 (items three and four; Kroenke et al., 2009), GAD‐7 (Spitzer et al., 2006) and DASS‐21 anxiety subscale (Henry & Crawford, 2005). The average score across items of a measure was *z*‐converted for each dataset to ensure consistency across datasets.

#### Trait loneliness

Baseline loneliness was assessed using the UCLA Loneliness Scale (Russell, 1996). Since different datasets used a different number of items (full 10‐item version or 3‐item version), the average score across items of a measure was *z*‐converted for each dataset to ensure consistency *across* datasets.

### Association between amount of time spent in social context and affect (daily dataset)

When testing the models examining the association between time spent in various social contexts and affect, three studies met the eligibility criteria, that is, assessed the amount of time in social context, rather than the binary state. Despite including more than three studies with daily diary design (see Table 1), most of them were included in momentary dataset, as they have assessed the state (yes/no), rather than the amount of time.

Of the included studies, one did not explicitly define the ‘public solitude’ group in the analysis of this dataset. Further, the ‘digitally mediated interactions when with others’ group was missing in one study. As a result, for the daily dataset, only three contexts were included: complete solitude, digitally mediated interactions when alone, and face‐to‐face interactions.

#### Daily minutes spent in complete solitude

The number of minutes participants spent in complete solitude on the day was recorded. If the amount of time was provided in hours, the number was converted to minutes.

#### Daily minutes spent engaging in digitally mediated interactions when alone

The number of minutes participants spent interacting with others digitally on the day. If the amount of time was provided in hours, the number was converted to minutes.

#### Daily minutes spent interacting face‐to‐face

The number of minutes participants spent interacting with others face‐to‐face during the day. If the amount of time was provided in hours, the number was converted to minutes.

### Momentary association between affect and social contexts (momentary dataset)

#### Momentary social context

Participants were asked to specify their social context in the moment: complete solitude, digitally mediated interactions when alone, public solitude, interacting face‐to‐face, digitally mediated interactions when with others.

### Analytic strategy

Two distinct datasets were constructed for the analyses. The daily dataset consisted of day‐level observations (Level 1, *N* = 6107) nested within participants (Level 2, *N* = 348) and captured the total daily amount of time spent in each available social context and corresponding daily affect. The momentary dataset consisted of momentary assessments (Level 1, *N* = 27,045) nested within participants (Level 2, *N* = 2642) and captured participants' momentary social context and affect at each assessment. To address between‐study differences in sample characteristics and mean affect levels, multilevel models included random intercepts for both participants and studies, thereby accounting for clustering at both levels and ensuring that effects were not conflated with study‐level baselines or sample‐specific differences.

Data were analysed using the nlme package (Pinheiro et al., 2023) in RStudio (RStudio Team, 2020). Given the nested nature of the data, that is, days and momentary assessments were nested within participants, multilevel modelling (Bryk & Raudenbush, 1992), also known as hierarchical linear modelling, was employed. This approach is particularly strong in such research as it allows for the analysis of data with complex dependency structures, such as repeated measures within individuals, and can provide more accurate estimates of effects at different levels.

The models were estimated as two‐level multilevel models, with repeated observations at Level 1 nested within participants at Level 2. Study was included as an additional random intercept rather than as a separate third level to account for between‐study differences in baseline affect and sample characteristics without estimating a full three‐level model. This approach allowed us to control for study‐level clustering while maintaining model parsimony, given the limited number of contributing studies.

Models were estimated using maximum likelihood (ML). ML estimation uses all available observations for variables included in a given model and can accommodate unbalanced data structures (e.g. differing numbers of observations per participant). Although ML estimation accommodates unbalanced repeated‐measures designs, nlme still excludes rows with missing values on variables included in a given model (including lagged predictors/outcomes), which can reduce the analysable sample for lagged models. Accordingly, models including baseline covariates were estimated on a reduced subset of participants.

For the daily dataset, multilevel models tested the extent to which the amount of time spent in complete solitude, engaging in digitally mediated interactions when alone, and interacting face‐to‐face on a given day (at Level 1), as well as age and extraversion (at Level 2), were associated with High‐Arousal Positive Affect, High‐Arousal Negative Affect, Low‐Arousal Positive Affect, Low‐Arousal Negative Affect and loneliness. The results reflect how spending longer or shorter periods in each social context is associated with affect.

For momentary dataset, multilevel models examined how the momentary state of being in one of the social contexts (coded as 1—complete solitude (reference category; not interacting face‐to‐face or digitally), 2—digitally mediated interactions when alone, 3—public solitude, 4—interacting face‐to‐face, 5—digitally mediated interactions when with others; Level 1), as well as age and extraversion (Level 2), were associated with High‐Arousal Positive Affect, High‐Arousal Negative Affect, Low‐Arousal Positive Affect, Low‐Arousal Negative Affect and loneliness. Coefficients for social context indicate differences relative to complete solitude, which was specified as the reference category. For example, a positive coefficient for face‐to‐face interaction reflects higher affect compared to complete solitude. Accordingly, a positive coefficient for a given social context indicates higher levels of the outcome relative to complete solitude, whereas a negative coefficient indicates lower levels.

In both datasets, baseline symptoms of depression, anxiety and loneliness were included as covariates in all models to adjust for individual differences in participants' affective and mental health profiles.

For each outcome, we aimed to estimate three models: (1) concurrent, (2) lagged context predicting subsequent affect and (3) lagged context controlling for the lagged outcome. Model 1 (concurrent) tests whether being in a given context is associated with affect at the same timepoint. Model 2 (lagged) tests whether being in a context predicts affect at the subsequent timepoint, providing a test of temporal ordering. Model 3 (lagged with prior outcome controlled, estimated using a first‐order autoregressive correlation structure at the participant level nested within study) provides the most stringent test, estimating whether context predicts change in affect beyond a person's own prior affective state. Together, they provide increasingly rigorous tests of temporal robustness.

Exploratory analyses for Model 1 (concurrent) examined whether results varied by day of the week, with models estimated twice: once with weekday treated as a categorical variable and once with weekday treated as a continuous variable. For Models 2 and 3 (lagged with autoregressive effects), it was not possible to include weekday in the analysis due to the structure of the data; therefore, weekday effects were not tested.

According to Cohen's (1988) guidelines for interpreting effect sizes, an effect size of 0.10 or smaller is generally considered small, and in this context, effect sizes close to zero (e.g. below 0.05) may be considered practically insignificant. Additionally, Funder and Ozer (2019) emphasize that statistically significant results with very small effect sizes can often have limited practical meaning. Therefore, we considered effect sizes below *b* = .05 to indicate negligible associations, even if they have reached significance.

### Sensitivity analyses

To evaluate whether results were dependent on the inclusion of baseline covariates, all models were re‐estimated excluding baseline symptoms of depression, anxiety and loneliness. This also allowed examination of the digitally mediated interactions within others' social context category, which contained 125 momentary observations but had no usable observations after listwise deletion in the main models due to missing covariate data. For the daily dataset, the model structure and estimation procedure were otherwise identical. For the momentary dataset, removing baseline covariates substantially increased the analysable sample; however, this resulted in convergence issues when fitting the autoregressive correlation structure (McNeish, 2017). Sensitivity models were therefore estimated without the AR(1) correlation, while retaining the fixed‐effects structure and random intercept specification. The fixed‐effects structure and random intercept specification were otherwise unchanged.

## RESULTS

### Intraclass correlation coefficient

The intraclass correlation (ICC) represents the percentage of total variance that is due to individual differences at Level 2. In the present analyses, the ICC reflects the proportion of variance attributable to between‐person differences, rather than between‐study differences, because participants constituted the highest modelled level. Variance associated with study‐level clustering was accounted for via random intercepts for study and is therefore not reflected in the reported ICC values. For example, an ICC of .60 indicates that 60% of the total variance in that outcome is attributable to stable between‐person differences, meaning that knowing a person's general level of affect accounts for a substantial portion of any given observation, while the remaining 40% reflects day‐to‐day or moment‐to‐moment fluctuation within individuals.

Null models (i.e. models with no predictors) were used to estimate the ICC. For the daily dataset, null models predicting five affective outcomes (High‐Arousal Positive Affect, High‐Arousal Negative Affect, Low‐Arousal Positive Affect, Low‐Arousal Negative Affect, Loneliness) yielded the ICC ranging from .51 to .65, indicating that between 51% and 65% of the variance reflects stable between‐person differences, whereas the remaining 35%–49% reflecting within‐person fluctuations. For the momentary dataset, null models predicting five affective outcomes (High‐Arousal Positive Affect, High‐Arousal Negative Affect, Low‐Arousal Positive Affect, Low‐Arousal Negative Affect, Loneliness) yielded ICCs ranging between .35 and .97, indicating that between 35% and 97% of the variance reflects stable between‐person differences, whereas the remaining 3%–65% reflects within‐person fluctuations. Thus, between‐person variance accounted for a substantial proportion of the total variance in both datasets, underscoring the need to analyse the data using multilevel modelling. See Supporting Information for the summary of ICCs for each outcome.

### Association between affect and amount of time spent in different social contexts (daily dataset)

Study authors and participant numbers were specified as cluster variables to account for the nested structure of the data.

Across outcomes, associations between time spent in different contexts and affect were consistently non‐significant or negligible in magnitude.

#### Loneliness (concurrent, model 1)

Time spent interacting face‐to‐face was statistically significantly associated with lower loneliness (*b* = −0.00, *SE* = 0.00, *t* = −5.01, *p* < .001); however, the association's effect size was close to zero. Time spent in digitally mediated interactions when alone (*b* = 0.00, *SE* = 0.00, *t* = 1.60, *p* = .110) and time spent in complete solitude (*b* = 0.00, *SE* = 0.00, *t* = 0.57, *p* = .566) were not statistically significantly associated with loneliness.

Extraversion (*b* = −0.06, *SE* = 0.07, *t* = −0.78, *p* = .435), age (*b* = −0.00, *SE* = 0.00, *t* = −1.38, *p* = .169), weekday (*b* = −0.01, *SE* = 0.01, *t* = −0.80, *p* = .425), baseline loneliness (*b* = −0.07, *SE* = 0.05, *t* = −1.45, *p* = .147) and baseline depression (*b* = 0.02, *SE* = 0.07, *t* = 0.23, *p* = .820) were not statistically significantly associated with loneliness. Baseline anxiety was statistically significantly associated with higher loneliness (*b* = 0.14, *SE* = 0.06, *t* = 2.34, *p* = .019).

#### Loneliness (lagged, model 2)

Lagged interacting face‐to‐face (*b* = −0.00, *SE* = 0.00, *t* = −0.14, *p* = .888), lagged digitally mediated interactions when alone (*b* = −0.00, *SE* = 0.00, *t* = −1.70, *p* = .088) and lagged time in complete solitude (*b* = 0.00, *SE* = 0.00, *t* = 0.21, *p* = .834) were not statistically significantly associated with loneliness.

Extraversion (*b* = −0.05, *SE* = 0.07, *t* = −0.74, *p* = .463), age (*b* = −0.00, *SE* = 0.00, *t* = −1.06, *p* = .289), weekday (*b* = −0.01, *SE* = 0.01, *t* = −1.30, *p* = .192), baseline loneliness (*b* = −0.06, *SE* = 0.04, *t* = −1.25, *p* = .213), baseline anxiety (*b* = 0.10, *SE* = 0.06, *t* = 1.57, *p* = .118) and baseline depression (*b* = 0.07, *SE* = 0.07, *t* = 1.02, *p* = .307) were not statistically significantly associated with loneliness.

#### High‐arousal negative affect (concurrent, model 1)

Time spent interacting face‐to‐face (*b* = −0.00, *SE* = 0.00, *t* = −0.10, *p* = .917), digitally mediated interactions when alone (*b* = 0.00, *SE* = 0.00, *t* = 1.75, *p* = .080) and in complete solitude (*b* = −0.00, *SE* = 0.00, *t* = −0.44, *p* = .656) was not statistically significantly associated with high‐arousal negative affect.

Extraversion (*b* = −0.02, *SE* = 0.07, *t* = −0.26, *p* = .793), age (*b* = −0.00, *SE* = 0.00, *t* = −1.41, *p* = .159), baseline loneliness (*b* = 0.04, *SE* = 0.05, *t* = 0.76, *p* = .447) and baseline depression (*b* = 0.01, *SE* = 0.07, *t* = 0.11, *p* = .911) were not statistically significantly associated with high‐arousal negative affect.

Baseline anxiety (*b* = 0.22, *SE* = 0.06, *t* = 3.51, *p* < .001) and weekday (*b* = −0.02, *SE* = 0.01, *t* = −3.35, *p* < .001) were statistically significantly associated with high‐arousal negative affect, although the association between weekday and high‐arousal negative affect was close to zero.

#### High‐arousal negative affect (lagged, model 2)

Lagged interacting face‐to‐face (*b* = −0.00, *SE* = 0.00, *t* = −1.48, *p* = .140), lagged digitally mediated interactions when alone (*b* = 0.00, *SE* = 0.00, *t* = 0.29, *p* = .774), lagged time in complete solitude (*b* = 0.00, *SE* = 0.00, *t* = 0.63, *p* = .528) were not statistically significantly associated with high‐arousal negative affect.

Extraversion (*b* = −0.03, *SE* = 0.07, *t* = −0.40, *p* = .689), age (*b* = −0.01, *SE* = 0.00, *t* = −1.61, *p* = .108), baseline loneliness (*b* = 0.01, *SE* = 0.05, *t* = 0.29, *p* = .768) and baseline depression (*b* = −0.01, *SE* = 0.07, *t* = −0.16, *p* = .877) were not statistically significantly associated with high‐arousal negative affect.

Baseline anxiety (*b* = 0.23, *SE* = 0.06, *t* = 3.53, *p* < .001) and weekday (*b* = −0.02, *SE* = 0.01, *t* = −2.10, *p* = .036) were statistically significantly associated with high‐arousal negative affect, although the association between weekday and high‐arousal negative affect was close to zero.

#### Low‐arousal positive affect (concurrent, model 1)

Time spent interacting face‐to‐face (*b* = 0.00, *SE* = 0.00, *t* = 1.53, *p* = .126), time spent in digitally mediated interactions when alone (*b* = −0.00, *SE* = 0.00, *t* = −0.29, *p* = .773) and time spent in complete solitude (*b* = −0.00, *SE* = 0.00, *t* = −0.60, *p* = .551) were not statistically significantly associated with low‐arousal positive affect.

Extraversion (*b* = 0.13, *SE* = 0.08, *t* = 1.62, *p* = .107), weekday (*b* = 0.01, *SE* = 0.01, *t* = 0.97, *p* = .334), baseline loneliness (*b* = −0.06, *SE* = 0.05, *t* = −1.26, *p* = .206), baseline anxiety (*b* = 0.09, *SE* = 0.07, *t* = 1.26, *p* = .208) and baseline depression (*b* = −0.11, *SE* = 0.08, *t* = −1.37, *p* = .172) were not statistically significantly associated with low‐arousal positive affect.

Age was statistically significantly associated with higher low‐arousal positive affect (*b* = 0.01, *SE* = 0.00, *t* = 2.65, *p* = .008), although the association was close to zero.

#### Low‐arousal positive affect (lagged, model 2)

Lagged interacting face‐to‐face (*b* = −0.00, *SE* = 0.00, *t* = −0.86, *p* = .392), lagged digitally mediated interactions when alone (*b* = −0.00, *SE* = 0.00, *t* = −0.09, *p* = .925) and lagged time in complete solitude (*b* = 0.00, *SE* = 0.00, *t* = 0.01, *p* = .991) were not statistically significantly associated with low‐arousal positive affect.

Extraversion (*b* = 0.14, *SE* = 0.08, *t* = 1.71, *p* = .090), baseline loneliness (*b* = −0.05, *SE* = 0.05, *t* = −0.92, *p* = .357) and baseline anxiety (*b* = 0.13, *SE* = 0.07, *t* = 1.85, *p* = .065) were not statistically significantly associated with low‐arousal positive affect.

Age (*b* = 0.01, *SE* = 0.00, *t* = 2.55, *p* = .011) and weekday (*b* = 0.02, *SE* = 0.01, *t* = 2.04, *p* = .042) were statistically significantly associated with higher low‐arousal positive affect, although the association was close to zero. Baseline depression was statistically significantly associated with lower low‐arousal positive affect (*b* = −0.18, *SE* = 0.08, *t* = −2.23, *p* = .026).

#### Low‐arousal negative affect (concurrent, model 1)

Time spent interacting face‐to‐face was statistically significantly associated with lower low‐arousal negative affect (*b* = −0.00, *SE* = 0.00, *t* = −4.66, *p* < .001), although the effect size was close to zero. Time spent in digitally mediated interactions when alone (*b* = −0.00, *SE* = 0.00, *t* = −0.79, *p* = .429) and time in complete solitude (*b* = −0.00, *SE* = 0.00, *t* = −0.74, *p* = .461) were not statistically significantly associated with low‐arousal negative affect.

Extraversion (*b* = −0.03, *SE* = 0.06, *t* = −0.50, *p* = .617), weekday (*b* = −0.00, *SE* = 0.00, *t* = −0.75, *p* = .452), baseline loneliness (*b* = −0.05, *SE* = 0.04, *t* = −1.37, *p* = .172) and baseline anxiety (*b* = 0.09, *SE* = 0.05, *t* = 1.83, *p* = .067) were not statistically significantly associated with low‐arousal negative affect.

Age was statistically significantly associated with lower low‐arousal negative affect (*b* = −0.01, *SE* = 0.00, *t* = −3.34, *p* < .001), although the effect size was close to zero. Baseline depression was statistically significantly associated with higher low‐arousal negative affect (*b* = 0.12, *SE* = 0.05, *t* = 2.21, *p* = .027).

#### Low‐arousal negative affect (lagged, model 2)

Lagged interacting face‐to‐face (*b* = −0.00, *SE* = 0.00, *t* = −1.25, *p* = .210), lagged digitally mediated interactions when alone (*b* = −0.00, *SE* = 0.00, *t* = −0.35, *p* = .728) and lagged time in complete solitude (*b* = 0.00, *SE* = 0.00, *t* = 0.59, *p* = .553) were not statistically significantly associated with low‐arousal negative affect.

Extraversion (*b* = −0.04, *SE* = 0.06, *t* = −0.71, *p* = .478), weekday (*b* = 0.00, *SE* = 0.01, *t* = 0.18, *p* = .860), baseline loneliness (*b* = −0.06, *SE* = 0.04, *t* = −1.60, *p* = .109) and baseline anxiety (*b* = 0.06, *SE* = 0.05, *t* = 1.14, *p* = .254) were not statistically significantly associated with low‐arousal negative affect. Age was statistically significantly associated with lower low‐arousal negative affect (*b* = −0.01, *SE* = 0.00, *t* = −2.89, *p* = .004), although the effect size was close to zero. Baseline depression was statistically significantly associated with higher low‐arousal negative affect (*b* = 0.17, *SE* = 0.06, *t* = 3.03, *p* = .002).

#### High‐arousal positive affect (concurrent, model 1)

Time spent interacting face‐to‐face was statistically significantly associated with high‐arousal positive affect (*b* = 0.00, *SE* = 0.00, *t* = 7.33, *p* < .001), although the effect size was close to zero. Time spent in digitally mediated interactions when alone was statistically significantly associated with high arousal positive affect (*b* = 0.00, *SE* = 0.00, *t* = 2.01, *p* = .045), although the effect size was close to zero. Time spent in complete solitude (*b* = −0.00, *SE* = 0.00, *t* = −1.86, *p* = .063) was not statistically significantly associated with high‐arousal positive affect.

Extraversion was statistically significantly associated with higher high‐arousal positive affect (*b* = 0.21, *SE* = 0.06, *t* = 3.54, *p* < .001). Age (*b* = 0.00, *SE* = 0.00, *t* = 0.88, *p* = .377), weekday (*b* = 0.01, *SE* = 0.00, *t* = 1.16, *p* = .248), baseline loneliness (*b* = 0.04, *SE* = 0.04, *t* = 1.05, *p* = .293), baseline anxiety (*b* = −0.04, *SE* = 0.05, *t* = −0.92, *p* = .359) and baseline depression (*b* = −0.01, *SE* = 0.05, *t* = −0.13, *p* = .898) were not statistically significantly associated with high‐arousal positive affect.

#### High‐arousal positive affect (lagged, model 2)

Lagged interacting face‐to‐face (*b* = 0.00, *SE* = 0.00, *t* = 0.20, *p* = .838), lagged digitally mediated interactions when alone (*b* = 0.00, *SE* = 0.00, *t* = 1.28, *p* = .199) and lagged time in complete solitude (*b* = 0.00, *SE* = 0.00, *t* = 0.45, *p* = .654) were not statistically significantly associated with subsequent high‐arousal positive affect.

Extraversion was statistically significantly associated with higher high‐arousal positive affect (*b* = 0.22, *SE* = 0.06, *t* = 3.67, *p* < .001). Age (*b* = 0.00, *SE* = 0.00, *t* = 0.56, *p* = .573), weekday (*b* = 0.01, *SE* = 0.01, *t* = 1.88, *p* = .060), baseline loneliness (*b* = 0.01, *SE* = 0.04, *t* = 0.36, *p* = .720), baseline anxiety (*b* = 0.00, *SE* = 0.05, *t* = 0.01, *p* = .990) and baseline depression (*b* = −0.06, *SE* = 0.06, *t* = −1.02, *p* = .310) were not statistically significantly associated with subsequent high‐arousal positive affect.

#### Sensitivity analyses

Sensitivity analyses were conducted to explore whether the results remain stable if baseline depression, anxiety and loneliness were removed. The results were identical in direction and significance to the ones reported above.

### Association between affect and momentary social contexts (momentary dataset)

#### Loneliness (concurrent, model 1)

Interacting face‐to‐face (*b* = 0.01, *SE* = 0.01, *t* = 0.49, *p* = .625), extraversion (*b* = 0.01, *SE* = 0.06, *t* = 0.13, *p* = .899), age (*b* = −0.00, *SE* = 0.01, *t* = −0.50, *p* = .619), baseline loneliness (*b* = 0.05, *SE* = 0.04, *t* = 1.51, *p* = .145), baseline anxiety (*b* = 0.06, *SE* = 0.08, *t* = 0.75, *p* = .462) and baseline depression (*b* = −0.06, *SE* = 0.06, *t* = −0.97, *p* = .344) were not statistically significantly associated with loneliness.

#### Loneliness (lagged, model 2)

Digitally mediated interactions when alone (*b* = 0.00, *SE* = 0.01, *t* = 0.47, *p* = .641), interacting face‐to‐face (*b* = −0.01, *SE* = 0.01, *t* = −0.76, *p* = .448), extraversion (*b* = −0.00, *SE* = 0.02, *t* = −0.10, *p* = .923), age (*b* = −0.00, *SE* = 0.00, *t* = −0.22, *p* = .829) and baseline depression (*b* = −0.02, *SE* = 0.02, *t* = −1.19, *p* = .244) were not statistically significantly associated with subsequent loneliness. Public solitude (*b* = 0.02, *SE* = 0.01, *t* = 2.41, *p* = .017), although the effect size was close to zero, was statistically significantly associated with higher subsequent loneliness. Baseline loneliness (*b* = 0.03, *SE* = 0.01, *t* = 2.04, *p* = .050), although the effect size was close to zero and baseline anxiety (*b* = 0.10, *SE* = 0.03, *t* = 3.70, *p* < .001) were statistically significantly associated with higher loneliness.

#### Loneliness (lagged + prior outcome, model 3)

Interacting face‐to‐face at the prior timepoint (*b* = −0.01, *SE* = 0.01, *t* = −1.09, *p* = .283), extraversion (*b* = −0.03, *SE* = 0.03, *t* = −1.17, *p* = .262), age (*b* = −0.00, *SE* = 0.00, *t* = −0.02, *p* = .988), baseline loneliness (*b* = −0.00, *SE* = 0.01, *t* = −0.29, *p* = .779), baseline anxiety (*b* = −0.01, *SE* = 0.04, *t* = −0.29, *p* = .773), baseline depression (*b* = 0.01, *SE* = 0.03, *t* = 0.48, *p* = .641) and prior loneliness (*b* = 0.01, *SE* = 0.08, *t* = 0.12, *p* = .903) were not statistically significantly associated with subsequent loneliness.

#### High‐arousal negative affect (concurrent, model 1)

Interacting face‐to‐face (*b* = −0.17, *SE* = 0.11, *t* = −1.65, *p* = .105), extraversion (*b* = 0.32, *SE* = 0.25, *t* = 1.27, *p* = .217), age (*b* = −0.00, *SE* = 0.02, *t* = −0.10, *p* = .923), baseline loneliness (*b* = 0.04, *SE* = 0.14, *t* = 0.26, *p* = .797) and baseline depression (*b* = −0.35, *SE* = 0.23, *t* = −1.51, *p* = .144) were not statistically significant. Baseline anxiety (*b* = 0.77, *SE* = 0.31, *t* = 2.46, *p* = .022) was statistically significantly associated with higher high‐arousal negative affect.

#### High‐arousal negative affect (lagged, model 2)

Digitally mediated interactions when alone (*b* = 0.05, *SE* = 0.08, *t* = 0.66, *p* = .508), public solitude (*b* = 0.03, *SE* = 0.08, *t* = 0.31, *p* = .755), interacting face‐to‐face (*b* = −0.04, *SE* = 0.09, *t* = −0.45, *p* = .656), extraversion (*b* = 0.18, *SE* = 0.14, *t* = 1.27, *p* = .212), age (*b* = 0.00, *SE* = 0.01, *t* = 0.03, *p* = .973), baseline loneliness (*b* = 0.02, *SE* = 0.08, *t* = 0.20, *p* = .842) and baseline depression (*b* = −0.17, *SE* = 0.10, *t* = −1.77, *p* = .087) were not statistically significant. Baseline anxiety (*b* = 0.66, *SE* = 0.16, *t* = 4.04, *p* < .001) was statistically significantly associated with higher high‐arousal negative affect.

#### High‐arousal negative affect (lagged + prior outcome, model 3)

Interacting face‐to‐face (*b* = −0.05, *SE* = 0.07, *t* = −0.75, *p* = .458), extraversion (*b* = 0.09, *SE* = 0.14, *t* = 0.64, *p* = .532), age (*b* = 0.02, *SE* = 0.01, *t* = 3.85, *p* = .002), baseline loneliness (*b* = −0.13, *SE* = 0.07, *t* = −2.03, *p* = .063), baseline anxiety (*b* = −0.18, *SE* = 0.21, *t* = −0.82, *p* = .427), baseline depression (*b* = 0.09, *SE* = 0.17, *t* = 0.57, *p* = .579) and prior high‐arousal negative affect (*b* = 0.22, *SE* = 0.07, *t* = 3.05, *p* = .005) were estimated; only prior affect and age were statistically significant, although the effect size for age was close to zero.

#### Low‐arousal positive affect (concurrent, model 1)

Interacting face‐to‐face (*b* = 0.06, *SE* = 0.11, *t* = 0.51, *p* = .613), extraversion (*b* = 0.15, *SE* = 0.31, *t* = 0.49, *p* = .632), age (*b* = 0.02, *SE* = 0.02, *t* = 0.77, *p* = .450), baseline loneliness (*b* = −0.03, *SE* = 0.17, *t* = −0.16, *p* = .872), baseline anxiety (*b* = −0.04, *SE* = 0.39, *t* = −0.11, *p* = .914) and baseline depression (*b* = 0.12, *SE* = 0.28, *t* = 0.43, *p* = .672) were not statistically significant.

#### Low‐arousal positive affect (lagged, model 2)

Digitally mediated interactions when alone (*b* = −0.11, *SE* = 0.08, *t* = −1.38, *p* = .170), public solitude (*b* = −0.00, *SE* = 0.08, *t* = −0.04, *p* = .970), extraversion (*b* = 0.18, *SE* = 0.22, *t* = 0.81, *p* = .424), age (*b* = 0.01, *SE* = 0.02, *t* = 0.57, *p* = .570), baseline loneliness (*b* = −0.12, *SE* = 0.13, *t* = −0.97, *p* = .339), baseline anxiety (*b* = 0.23, *SE* = 0.26, *t* = 0.95, *p* = .352) and baseline depression (*b* = 0.02, *SE* = 0.16, *t* = 0.13, *p* = .895) were not statistically significant. Interacting face‐to‐face (*b* = −0.18, *SE* = 0.09, *t* = −2.06, *p* = .041) was statistically significantly associated with lower subsequent low‐arousal positive affect. Extraversion, age and baseline covariates were not statistically significant.

#### Low‐arousal positive affect (lagged + prior outcome, model 3)

Interacting face‐to‐face (*b* = −0.04, *SE* = 0.10, *t* = −0.43, *p* = .669), extraversion (*b* = −0.08, *SE* = 0.31, *t* = −0.24, *p* = .811), age (*b* = −0.02, *SE* = 0.02, *t* = −0.86, *p* = .406), baseline loneliness (*b* = 0.07, *SE* = 0.15, *t* = 0.48, *p* = .637), baseline anxiety (*b* = 0.30, *SE* = 0.42, *t* = 0.72, *p* = .485) and baseline depression (*b* = 0.40, *SE* = 0.29, *t* = 1.37, *p* = .194) were not statistically significant. Prior low‐arousal positive affect (*b* = 0.26, *SE* = 0.10, *t* = 2.78, *p* = .009) was statistically significantly associated with subsequent low‐arousal positive affect.

#### Low‐arousal negative affect (concurrent, model 1)

Interacting face‐to‐face (*b* = 0.02, *SE* = 0.03, *t* = 0.58, *p* = .563), extraversion (*b* = −0.04, *SE* = 0.09, *t* = −0.42, *p* = .676), age (*b* = 0.00, *SE* = 0.01, *t* = 0.52, *p* = .606), baseline loneliness (*b* = 0.05, *SE* = 0.05, *t* = 1.11, *p* = .278), baseline anxiety (*b* = 0.13, *SE* = 0.10, *t* = 1.26, *p* = .220) and baseline depression (*b* = −0.05, *SE* = 0.08, *t* = −0.60, *p* = .554) were not statistically significantly associated with low‐arousal negative affect.

#### Low‐arousal negative affect (lagged, model 2)

Digitally mediated interactions when alone (*b* = 0.04, *SE* = 0.02, *t* = 1.53, *p* = .128), public solitude (*b* = 0.03, *SE* = 0.02, *t* = 1.17, *p* = .245), interacting face‐to‐face (*b* = −0.00, *SE* = 0.03, *t* = −0.14, *p* = .889), extraversion (*b* = −0.03, *SE* = 0.05, *t* = −0.68, *p* = .503), age (*b* = 0.00, *SE* = 0.01, *t* = 0.10, *p* = .921), baseline loneliness (*b* = 0.04, *SE* = 0.03, *t* = 1.36, *p* = .184) and baseline depression (*b* = −0.01, *SE* = 0.04, *t* = −0.15, *p* = .883) were not statistically significantly associated with low‐arousal negative affect. Baseline anxiety (*b* = 0.13, *SE* = 0.06, *t* = 2.25, *p* = .032) was statistically significantly associated with higher low‐arousal negative affect.

#### Low‐arousal negative affect (lagged + prior outcome, model 3)

Interacting face‐to‐face (*b* = −0.01, *SE* = 0.03, *t* = −0.28, *p* = .781), extraversion (*b* = −0.06, *SE* = 0.06, *t* = −0.98, *p* = .346), age (*b* = 0.00, *SE* = 0.00, *t* = 0.29, *p* = .776), baseline loneliness (*b* = −0.02, *SE* = 0.03, *t* = −0.76, *p* = .458), baseline anxiety (*b* = 0.03, *SE* = 0.08, *t* = 0.34, *p* = .740) and baseline depression (*b* = 0.02, *SE* = 0.06, *t* = 0.27, *p* = .792) were not statistically significantly associated with low‐arousal negative affect. Prior low‐arousal negative affect (*b* = 0.34, *SE* = 0.09, *t* = 3.68, *p* = .001) was statistically significantly associated with subsequent low‐arousal negative affect.

#### High‐arousal positive affect (concurrent, model 1)

Interacting face‐to‐face (*b* = 0.06, *SE* = 0.22, *t* = 0.28, *p* = .779), extraversion (*b* = 0.20, *SE* = 0.51, *t* = 0.38, *p* = .706), baseline anxiety (*b* = −0.52, *SE* = 0.63, *t* = −0.83, *p* = .418), baseline depression (*b* = 0.15, *SE* = 0.47, *t* = 0.32, *p* = .754) and baseline loneliness (*b* = −0.53, *SE* = 0.28, *t* = −1.92, *p* = .068) were not statistically significantly associated with high‐arousal positive affect. Age (*b* = −0.08, *SE* = 0.04, *t* = −2.19, *p* = .040) was statistically significantly associated with lower high‐arousal positive affect.

#### High‐arousal positive affect (lagged, model 2)

Digitally mediated interactions when alone (*b* = 0.07, *SE* = 0.17, *t* = 0.42, *p* = .673), public solitude (*b* = 0.14, *SE* = 0.17, *t* = 0.83, *p* = .409), extraversion (*b* = −0.19, *SE* = 0.37, *t* = −0.50, *p* = .617), baseline loneliness (*b* = 0.05, *SE* = 0.21, *t* = 0.24, *p* = .812), baseline anxiety (*b* = −0.37, *SE* = 0.43, *t* = −0.86, *p* = .396) and baseline depression (*b* = 0.24, *SE* = 0.26, *t* = 0.91, *p* = .371) were not statistically significantly associated with subsequent high‐arousal positive affect. Interacting face‐to‐face (*b* = 0.42, *SE* = 0.21, *t* = 2.04, *p* = .043) and age (*b* = −0.11, *SE* = 0.04, *t* = −2.81, *p* = .008) were statistically significantly associated with higher subsequent high‐arousal positive affect.

#### High‐arousal positive affect (lagged, model 2, moderation analysis)

Lagged face‐to‐face time (*b* = 0.00, *SE* = 0.00, *t* = 0.17, *p* = .867), lagged digitally mediated interactions when alone (*b* = 0.00, *SE* = 0.00, *t* = 1.35, *p* = .178) and lagged time in complete solitude (*b* = 0.00, *SE* = 0.00, *t* = 0.91, *p* = .365) were not statistically significantly associated with subsequent high‐arousal positive affect. Baseline loneliness (*b* = 0.01, *SE* = 0.04, *t* = 0.31, *p* = .756), baseline anxiety (*b* = 0.00, *SE* = 0.05, *t* = −0.02, *p* = .986), baseline depression (*b* = −0.06, *SE* = 0.06, *t* = −1.00, *p* = .319), age (*b* = 0.00, *SE* = 0.00, *t* = 0.59, *p* = .556) and weekday (*b* = 0.01, *SE* = 0.01, *t* = 1.88, *p* = .060) were not statistically significantly associated with subsequent high‐arousal positive affect. Extraversion was statistically significantly associated with higher subsequent high‐arousal positive affect (*b* = 0.43, *SE* = 0.09, *t* = 4.54, *p* < .001).

The interaction between lagged face‐to‐face time and extraversion (*b* = 0.00, *SE* = 0.00, *t* = −0.19, *p* = .851) and the interaction between lagged digitally mediated interactions when alone and extraversion (*b* = 0.00, *SE* = 0.00, *t* = −0.37, *p* = .713) were not statistically significant. The interaction between lagged time in complete solitude and extraversion was statistically significant (*b* = −0.00, *SE* = 0.00, *t* = −2.91, *p* = .004), although the effect size was close to zero.

#### High‐arousal positive affect (lagged + prior outcome, model 3, moderation)

Interacting face‐to‐face (*b* = 0.47, *SE* = 0.28, *t* = 1.72, *p* = .095), extraversion (*b* = 1.20, *SE* = 0.60, *t* = 1.99, *p* = .068), age (*b* = −0.03, *SE* = 0.03, *t* = −1.05, *p* = .311), baseline loneliness (*b* = −0.45, *SE* = 0.30, *t* = −1.49, *p* = .161), baseline anxiety (*b* = −0.23, *SE* = 0.90, *t* = −0.26, *p* = .801) and baseline depression (*b* = −0.68, *SE* = 0.65, *t* = −1.04, *p* = .315) were not statistically significantly associated with high‐arousal positive affect. Prior high‐arousal positive affect (*b* = 0.45, *SE* = 0.15, *t* = 3.00, *p* = .005) was statistically significantly associated with subsequent high‐arousal positive affect.

See Supporting Information for the full set of results.

#### Sensitivity analyses

Sensitivity analyses examined the results without the inclusion of baseline covariates (depression, anxiety, loneliness). Sensitivity models were estimated without the AR(1) correlation. The fixed‐effects structure and random intercept specification were otherwise unchanged. Sensitivity analysis yielded a largely similar pattern of momentary associations. The key differences are summarized below.

For High‐Arousal Positive Affect, relative to complete solitude, concurrent affect (Model 1) was higher in digitally mediated interactions when alone (*b* = 0.38, *p* < .001), face‐to‐face interactions (*b* = 0.91, *p* < .001) and digitally mediated interactions when with others (*b* = 0.34, *p* = .002) contexts. In lagged models (Model 2), only prior face‐to‐face interactions predicted higher subsequent High‐Arousal Positive Affect (*b* = 0.21, *p* = .0002), but this effect did not persist after controlling for prior High‐Arousal Positive Affect (*b* = 0.04, *p* = .557; prior affect *b* = 0.21, *p* < .001).

For Low‐Arousal Negative Affect, concurrent affect (Model 1) was lower in digital interactions when alone (*b* = −0.14, *p* < .001) and face‐to‐face (*b* = −0.24, *p* < .001) contexts compared to complete solitude; lagged face‐to‐face interactions (Model 2) predicted lower subsequent affect (*b* = −0.06, *p* = .003) compared to complete solitude.

For High‐Arousal Negative Affect, concurrent affect was lower in face‐to‐face context compared to complete solitude (*b* = −0.10, *p* = .024).

For loneliness, all contexts showed lower concurrent loneliness compared to complete solitude (Model 2): digitally mediated interactions when alone (*b =* −0.16, *p* < .001), public solitude (*b* = −0.10, *p* < .001), face‐to‐face interactions (*b* = −0.40, *p* < .001) and digitally mediated interactions when with others (*b* = −0.21, *p* < .001). Prospectively (Model 2), only prior face‐to‐face interactions predicted lower subsequent loneliness (*b* = −0.08, *p* = .0001), which was attenuated after controlling for prior loneliness (*b * = −0.02, *p* = .347; prior loneliness *b* = 0.15, *p* < .001).

## DISCUSSION

The current research presents a secondary analysis of diary studies that examined links between everyday social context, extraversion, age, loneliness and affect (operationalized on the dimensions of valence and arousal). Analyses comparing solitudinal and social contexts revealed several notable patterns. First, our daily analyses showed no substantive effects of time spent in different contexts on affective outcomes. However, momentary analyses identified that following face‐to‐face interactions, people experienced less low‐arousal but more high‐arousal positive affect. This finding highlights the importance of examining specific affect dimensions rather than treating positive and negative affect as unitary constructs. Second, higher baseline anxiety was associated with increased low‐arousal negative affect (daily dataset) as well as loneliness and high‐arousal negative affect (daily and momentary datasets). Third, in the daily dataset, baseline depression was associated with decreased low‐arousal positive affect and increased low‐arousal negative affect. Fourth, high‐arousal positive affect was positively related to extraversion in the daily dataset, while negatively related to age. Neither extraversion nor age moderated the relationship between social context and affect, suggesting that the contextual effects observed were not substantially shaped by these individual differences.

Spending longer periods of time in complete solitude was not associated with any affective outcomes in the main models, with effects being non‐significant and effect sizes very close to zero. Thus, we did not find evidence for one aspect of the *deactivation effect* frequently identified in solitude research (e.g. Bradshaw et al., 2025; Nguyen et al., 2018; Weinstein et al., 2023), in which individuals report greater calm, or low‐arousal positive affect, after time spent in solitude. Sensitivity analysis did indicate that complete solitude was a context associated with increased loneliness, compared to all other examined contexts; however, no affective benefits of solitude were observed.

Our results did not indicate that time spent in social interaction had cumulative associations with affect. Researchers have previously reported that while social interactions can bring a positive well‐being boost, time spent with others is beneficial only to a certain point, beyond which continuing social interactions do not confer any additional gains (Kushlev et al., 2018; Ren et al., 2022). Nevertheless, consistent with previous research (Bernstein et al., 2018), we did find that, compared to being in complete solitude, face‐to‐face interactions were associated with subsequent increases in high‐arousal positive affect in the momentary lagged model (although this effect did not remain significant when controlling for prior high‐arousal positive affect). Here, emotional contagion may explain why being around others may be associated with increases in both affective valence and arousal, as people often ‘catch’ the emotions of those around them, adopting a similar mood or emotional state (Hatfield et al., 1993; Qureshi et al., 2024). This phenomenon could partly account for the boost in well‐being seen in social contexts, even in brief encounters.

Sensitivity analysis in the momentary dataset additionally indicated that face‐to‐face interactions were linked to decreased low‐arousal negative affect, while digital interactions (both when alone and with others) were associated with increased high‐arousal positive affect. This shows that any interaction, even the one that is not in person, may provide a boost to a person's well‐being, and that this boost may occur regardless of the duration of these interactions. Yet, the mere presence of others is insufficient and may even harm well‐being if one is not actively interacting with them, as indicated by our finding from sensitivity analysis in the momentary dataset that spending time in public solitude may be associated with loneliness (compared to complete solitude). In sum, the study showed that interactions with others were associated with affective benefits; however, their mere presence is not sufficient and may produce opposite effects.

Altogether, the current results suggest that social participation plays an important role in high‐arousal positive affect, implying that interventions promoting social engagement could enhance well‐being. Further, our study demonstrated the importance of accounting for individual differences when examining the association between social context and well‐being. For example, the finding that increased extraversion is associated with increased high‐arousal positive affect in both daily and momentary datasets (although not in the concurrent model in the momentary dataset) refines previous research linking extraversion to well‐being more broadly (Costa & McCrae, 1980; Margolis & Lyubomirsky, 2020). This may be due to extraverts having more social interactions, which our study showed were also linked to increases in high‐arousal positive affect. This aligns with the social participation hypothesis, which suggests that social interactions, particularly in‐person interactions, are important for well‐being (Lucas et al., 2008; Oerlemans & Bakker, 2014).

### Limitations

The study, and the daily dataset in particular, largely revealed no associations between social contexts and affect. Two broad explanations are possible: either robust context‐affect associations do not exist at this level of aggregation, or methodological features of the available data precluded their detection. Below, we summarize the limitations of the study that may have contributed to the observed null results.

#### Limitations of diary studies

The daily diary design may have limited our ability to detect affective consequences of social context. Affective responses to social experiences may be short‐lived and dissipate within hours, meaning that aggregating experiences across an entire day could obscure transient but meaningful effects. Thus, the absence of strong daily associations should not necessarily be interpreted as evidence that social context is unrelated to affect, but rather that it is a weaker predictor of affect when other factors not considered at this study are at play.

In a similar vein, diary studies are correlational in nature. While findings suggested that high‐arousal positive affect increased following face‐to‐face interactions, it is equally plausible that individuals select social contexts based on their emotional state (Elmer, 2021; Quoidbach et al., 2019). For instance, individuals experiencing positive affect may be more motivated to engage in social interactions, while those experiencing low‐arousal negative affect (e.g. sadness) may be more likely to withdraw into solitude.

Further, diary studies introduce compliance issues. In our study, compliance rates varied across the contributing studies, with some daily diary protocols showing relatively low completion rates. Because diary and momentary assessment data rely on self‐report without external verification, selective compliance could be at play here. For instance, participants may have been less likely to report during particularly stressful, socially intense or busy periods. Although multilevel modelling accommodates unbalanced data structures, it does not correct for non‐random missingness. Reduced or selective compliance may therefore have attenuated observable associations between social context and affect.

#### Combination of affect measures

The harmonization of affect measures across studies required mapping different instruments (see Table 1) onto a common high‐low‐arousal by positive–negative valence framework. While this enabled integrative analyses across multiple datasets, it necessarily imposed a shared structure on measures that were originally developed with differing conceptual and psychometric properties. Therefore, a limitation of the present work is that affect was assessed using a range of scale types across studies, including unipolar and bipolar formats, different response ranges and both single‐ and multi‐item measures (see Table 1). Although we addressed this by reverse‐scoring bipolar items where needed and z‐standardizing all measures to place them on a common metric, differences in item wording and response format may still contribute to measurement variability. This heterogeneity could attenuate effect sizes or introduce noise into cross‐study comparisons.

#### Omission of situational characteristics

Further, as included studies did not consistently measure the setting one was in, the current research misses important nuances about how the context of interaction (e.g. work meeting versus a party) may be associated with affect. This may account for largely null results reported in this study, and further studies should therefore explore this association.

We also did not examine the quality of social interactions or who people were interacting with, factors that may be important for social context‐affect associations. Additionally, many other individual differences reflecting cultural, personality and background factors, as well as short‐term antecedents of life's social and solitude contexts, may play an important role (see, e.g. Orben & Przybylski, 2019 for this point in the context of digitally mediated interactions).

Next, the present research did not examine qualitative characteristics of social interactions, such as the closeness of the relationship, perceived support or conflict, voluntariness or the purpose of the interaction (e.g. work meeting vs social gathering). Social settings are inherently heterogeneous and affective consequences may depend more strongly on these qualitative features than on broad modality categories such as ‘face‐to‐face’ or ‘digitally mediated’. These characteristics were not consistently measured across studies; however, the omission of these characteristics may help explain the largely null findings in the daily models and the attenuation of effects in prospective models.

Furthermore, our research did not account for social context where a mixture of face‐to‐face and digital interactions is happening simultaneously, that is, being in the presence of others, interacting with them face‐to‐face, while also interacting with others digitally (e.g. a group video call where participants are both in‐person and remote). This differs from the ‘digitally mediated interactions’ context included in this study, as the included context considered being in the physical presence of others without interacting face‐to‐face, but interacting digitally (e.g. being in a café by yourself while messaging a friend). In a similar vein, due to the nature of included datasets, the study did not differentiate between verbal digital interactions (such as talking on the phone) and non‐verbal ones (such as texting). The quality of these interactions may be different and lead to different affective responses, which should be examined in future research.

Finally, some of the data included in the analysis was collected during the COVID‐19 pandemic, a unique time when social dynamics were different and people were spending more time in complete solitude or interacting digitally than interacting face‐to‐face, potentially skewing affective response.

#### Lab identification process

The search terms used to identify active research groups were ‘solitude’ OR ‘alone’, covering the period 2012–2022, with loneliness‐focused papers excluded during screening. However, the number of records retrieved and the stopping rule were not recorded. The search was supplemented by the last author's knowledge of the field; consequently, the identification process cannot be fully replicated. We acknowledge this limitation and encourage future research to conduct more systematic and fully documented searches of this literature.

Despite these limitations, the present study provides evidence for robust concurrent associations between momentary face‐to‐face interactions and affect, showing that following face‐to‐face interactions, people experienced less low‐arousal but more high‐arousal positive affect. Future research would benefit from designs that capture qualitative features of interactions, employ higher compliance monitoring and examine shorter temporal lags to better isolate causal processes linking social context and affect. If replicated, these findings may inform the design and implementation of clinical intervention and prevention efforts for enhancing well‐being.

## AUTHOR CONTRIBUTIONS

**Anna Tovmasyan:** Conceptualization; investigation; writing – original draft; methodology; validation; visualization; writing – review and editing; software; formal analysis; project administration; data curation; resources. **Denethri Gamagedara:** Writing – review and editing; data curation. **Kou Murayama:** Writing – review and editing; supervision. **Julie Bowker:** Writing – review and editing; resources. **Robert Coplan:** Writing – review and editing; resources. **Jennifer Lay:** Writing – review and editing; resources. **Thuy‐vy Nguyen:** Writing – review and editing; resources. **Dongning Ren:** Writing – review and editing; resources. **Olga Stavrova:** Writing – review and editing; resources. **Virginia Thomas:** Writing – review and editing; resources. **Dwight Tse:** Writing – review and editing; resources. **Netta Weinstein:** Conceptualization; writing – review and editing; supervision; funding acquisition.

## FUNDING INFORMATION

This article was funded by the European Research Council, grant number 85190_SOAR.

## CONFLICT OF INTEREST STATEMENT

The authors declare no conflicts of interest.

## Supporting information


**Data S1.** bjso70124‐sup‐0001‐Supinfo1.docx.

## Data Availability

The methods and results of the original project, as well as datasets and analysis scripts for the presented project, can be found on https://osf.io/6deg4/?view_only=fe5bd431e6264d3d89f7b2cee971634a.
