# Supplementary material for: Predictors of affect across social contexts: An integration of existing diary datasets
Source: Br J Soc Psychol. 2026 Aug 14;65(4):e70124. doi: 10.1111/bjso.70124 (PMC13474902; doi:10.1111/bjso.70124)
Supplement: Supplementary file 1 [file BJSO-65-0-s001.docx]

**Table 2. Intraclass Correlation Coefficients**

| **Affective Outcome** | **Daily dataset ICC** | **Momentary dataset ICC** |
| --- | --- | --- |
| **High Arousal Positive Affect** | .51 | .36 |
| **High Arousal Negative Affect** | .55 | .60 |
| **Low Arousal Positive Affect** | .65 | .35 |
| **Low Arousal Negative Affect** | .65 | .85 |
| **Loneliness** | .64 | .97 |

## **Table 3**

## **Fixed effects predicting Loneliness**

### **Daily dataset - Loneliness (Concurrent, Model 1)**

| **Predictor** | ***b*** | ***SE*** | ***t*** | ***p*** |
| --- | --- | --- | --- | --- |
| **Intercept** | -0.24 | 0.21 | -1.16 | 0.246 |
| **Time Spent Interacting face-to-face** | -0.00 | 0.00 | -5.01 | < .001 |
| **Time Spent in Digitally-Mediated Interactions when Alone** | 0.00 | 0.00 | 1.60 | 0.110 |
| **Minutes in Complete Solitude** | 0.00 | 0.00 | 0.57 | 0.566 |
| **Extraversion** | -0.06 | 0.07 | -0.78 | 0.435 |
| **Age** | -0.00 | 0.00 | -1.38 | 0.169 |
| **Weekday** | -0.01 | 0.01 | -0.80 | 0.425 |
| **Baseline Loneliness** | -0.07 | 0.05 | -1.45 | 0.147 |
| **Baseline Anxiety** | 0.14 | 0.06 | 2.34 | 0.019 |
| **Baseline Depression** | 0.02 | 0.07 | 0.23 | 0.820 |

### **Daily dataset - Loneliness (Lagged, Model 2)**

| **Predictor** | ***b*** | ***SE*** | ***t*** | ***p*** |
| --- | --- | --- | --- | --- |
| **Intercept** | -0.21 | 0.21 | -0.98 | 0.327 |
| **Time Spent Interacting face-to-face (lagged)** | -0.00 | 0.00 | -0.14 | 0.888 |
| **Time Spent in Digitally-Mediated Interactions when Alone (lagged)** | -0.00 | 0.00 | -1.70 | 0.088 |
| **Minutes in Complete Solitude (lagged)** | 0.00 | 0.00 | 0.21 | 0.834 |
| **Extraversion** | -0.05 | 0.07 | -0.74 | 0.463 |
| **Age** | -0.00 | 0.00 | -1.06 | 0.289 |
| **Weekday** | -0.01 | 0.01 | -1.30 | 0.192 |
| **Baseline Loneliness** | -0.06 | 0.04 | -1.25 | 0.213 |
| **Baseline Anxiety** | 0.10 | 0.06 | 1.57 | 0.118 |
| **Baseline Depression** | 0.07 | 0.07 | 1.02 | 0.307 |

### **Momentary dataset - Loneliness (Concurrent, Model 1)**

| **Predictor** | ***b*** | ***SE*** | ***t*** | ***p*** |
| --- | --- | --- | --- | --- |
| **Intercept** | -1.54 | 0.11 | -13.69 | <.001 |
| **Interacting face-to-face** | 0.01 | 0.01 | 0.12 | .625 |
| **Extraversion** | 0.01 | 0.06 | 0.12 | .899 |
| **Age** | -0.00 | 0.01 | 1.51 | .619 |
| **Baseline Loneliness** | 0.05 | 0.04 | 1.51 | .145 |
| **Baseline Anxiety** | 0.06 | 0.08 | 0.75 | .462 |
| **Baseline Depression** | -0.06 | 0.06 | -0.97 | .344 |

### **Momentary dataset - Loneliness (Lagged, Model 2)**

| **Predictor** | ***b*** | ***SE*** | ***t*** | ***p*** |
| --- | --- | --- | --- | --- |
| **Intercept** | -1.59 | 0.05 | -33.16 | <.011 |
| **Digitally-mediated interactions when alone (prior)** | 0.00 | 0.01 | 0.47 | .641 |
| **Public solitude (prior)** | 0.02 | 0.01 | 2.41 | .017 |
| **Interacting face-to-face (prior)** | -0.01 | 0.01 | -0.76 | .448 |
| **Extraversion** | -0.00 | 0.02 | -0.10 | .923 |
| **Age** | -0.00 | 0.00 | -0.22 | .829 |
| **Baseline Loneliness** | 0.03 | 0.01 | 2.04 | .050 |
| **Baseline Anxiety** | 0.10 | 0.03 | 3.70 | < .001 |
| **Baseline Depression** | -0.02 | 0.02 | -1.19 | .244 |

### **Daily dataset - Loneliness (Lagged + prior outcome, Model 3)**

| **Predictor** | ***b*** | ***SE*** | ***t*** | ***p*** |
| --- | --- | --- | --- | --- |
| **Intercept** | -1.56 | 0.13 | -12.23 | <.001 |
| **Face-to-face (prior)** | -0.01 | 0.01 | -1.09 | .283 |
| **Extraversion** | -0.03 | 0.03 | -1.17 | .262 |
| **Age** | -0.00 | 0.00 | -0.02 | .988 |
| **Baseline Loneliness** | -0.00 | 0.01 | -0.29 | .779 |
| **Baseline Anxiety** | -0.01 | 0.04 | -0.29 | .773 |
| **Baseline Depression** | 0.01 | 0.03 | 0.48 | .641 |
| **Prior Loneliness** | 0.01 | 0.08 | 0.12 | .903 |

## **Table 4 Fixed effects predicting High Arousal Negative Affect (HANA)**

### **Daily dataset - HANA (Concurrent, Model 1)**

| **Predictor** | ***b*** | ***SE*** | ***t*** | ***p*** |
| --- | --- | --- | --- | --- |
| **Intercept** | 0.12 | 0.21 | 0.56 | 0.573 |
| **Time Spent Interacting face-to-face** | -0.00 | 0.00 | -0.10 | 0.917 |
| **Time Spent in Digitally-Mediated Interactions when Alone** | 0.00 | 0.00 | 1.75 | 0.080 |
| **Minutes in Complete Solitude** | -0.00 | 0.00 | -0.44 | 0.656 |
| **Extraversion** | -0.02 | 0.07 | -0.26 | 0.793 |
| **Age** | -0.00 | 0.00 | -1.41 | 0.159 |
| **Weekday** | -0.02 | 0.01 | -3.35 | < .001 |
| **Baseline Loneliness** | 0.04 | 0.05 | 0.76 | 0.447 |
| **Baseline Anxiety** | 0.22 | 0.06 | 3.51 | < .001 |
| **Baseline Depression** | 0.01 | 0.07 | 0.11 | 0.911 |

### **Daily dataset - HANA (Lagged, Model 2)**

| **Predictor** | ***b*** | ***SE*** | ***t*** | ***p*** |
| --- | --- | --- | --- | --- |
| **Intercept** | 0.02 | 0.21 | 0.12 | 0.908 |
| **Time Spent Interacting face-to-face (lagged)** | -0.00 | 0.00 | -1.48 | 0.140 |
| **Time Spent in Digitally-Mediated Interactions when Alone (lagged)** | 0.00 | 0.00 | 0.29 | 0.774 |
| **Minutes in Complete Solitude (lagged)** | 0.00 | 0.00 | 0.63 | 0.528 |
| **Extraversion** | -0.03 | 0.07 | -0.40 | 0.689 |
| **Age** | -0.01 | 0.00 | -1.61 | 0.108 |
| **Weekday** | -0.02 | 0.01 | -2.10 | 0.036 |
| **Baseline Loneliness** | 0.01 | 0.05 | 0.29 | 0.768 |
| **Baseline Anxiety** | 0.23 | 0.06 | 3.53 | < .001 |
| **Baseline Depression** | -0.01 | 0.07 | -0.16 | 0.877 |

### **Momentary dataset - HANA (Concurrent, Model 1)**

| **Predictor** | ***b*** | ***SE*** | ***t*** | ***p*** |
| --- | --- | --- | --- | --- |
| **Intercept** | -0.14 | 0.41 | -0.33 | .739 |
| **Interacting face-to-face** | -0.17 | 0.11 | -1.65 | .105 |
| **Extraversion** | 0.32 | 0.25 | 1.27 | .217 |
| **Age** | -0.00 | 0.02 | -0.10 | .923 |
| **Baseline Loneliness** | 0.04 | 0.14 | 0.26 | .797 |
| **Baseline Anxiety** | 0.77 | 0.31 | 2.46 | .022 |
| **Baseline Depression** | -0.35 | 0.23 | -1.51 | .144 |

### **Momentary dataset - HANA (Lagged, Model 2)**

| **Predictor** | ***b*** | ***SE*** | ***t*** | ***p*** |
| --- | --- | --- | --- | --- |
| **Intercept** | -0.29 | 0.30 | -0.99 | .322 |
| **Digitally-mediated interactions when alone (prior)** | 0.05 | 0.08 | 0.66 | .508 |
| **Public solitude (prior)** | 0.03 | 0.08 | 0.31 | .754 |
| **Interacting face-to-face (prior)** | -0.04 | 0.09 | -0.45 | .656 |
| **Extraversion** | 0.18 | 0.14 | 1.27 | .212 |
| **Age** | 0.00 | 0.01 | 0.03 | .973 |
| **Baseline Loneliness** | 0.02 | 0.08 | 0.20 | .842 |
| **Baseline Anxiety** | 0.66 | 0.16 | 4.04 | < .001 |
| **Baseline Depression** | -0.17 | 0.10 | -1.77 | .087 |

### **Momentary dataset – HANA (Lagged + prior outcome, Model 3)**

| **Predictor** | ***b*** | ***SE*** | ***t*** | ***p*** |
| --- | --- | --- | --- | --- |
| **Intercept** | -0.61 | 0.17 | -3.59 | .001 |
| **Face-to-face (prior)** | -0.05 | 0.07 | -0.75 | .458 |
| **Extraversion** | 0.09 | 0.14 | 0.64 | .532 |
| **Age** | 0.02 | 0.01 | 3.85 | .002 |
| **Baseline Loneliness** | -0.13 | 0.07 | -2.03 | .063 |
| **Baseline Anxiety** | -0.18 | 0.21 | -0.82 | .427 |
| **Baseline Depression** | 0.09 | 0.17 | 0.57 | .579 |
| **Prior HANA** | 0.22 | 0.07 | 3.05 | .005 |

## **Table 5 Fixed effects predicting Low Arousal Positive Affect (LAPA)**

### **Daily dataset - LAPA (Concurrent, Model 1)**

| **Predictor** | ***b*** | ***SE*** | ***t*** | ***p*** |
| --- | --- | --- | --- | --- |
| **Intercept** | 1.08 | 0.23 | 4.63 | < .001 |
| **Time Spent Interacting face-to-face** | 0.00 | 0.00 | 1.53 | 0.126 |
| **Time Spent in Digitally-Mediated Interactions when Alone** | -0.00 | 0.00 | -0.60 | 0.551 |
| **Minutes in Complete Solitude** | -0.00 | 0.00 | -0.29 | 0.773 |
| **Extraversion** | 0.13 | 0.08 | 1.62 | 0.107 |
| **Age** | 0.01 | 0.00 | 2.65 | 0.008 |
| **Weekday** | 0.01 | 0.01 | 0.97 | 0.334 |
| **Baseline Loneliness** | -0.06 | 0.05 | -1.26 | 0.206 |
| **Baseline Anxiety** | 0.09 | 0.07 | 1.26 | 0.208 |
| **Baseline Depression** | -0.11 | 0.08 | -1.37 | 0.172 |

### **Daily dataset – LAPA (Lagged, Model 2)**

| **Predictor** | ***b*** | ***SE*** | ***t*** | ***p*** |
| --- | --- | --- | --- | --- |
| **Intercept** | 1.00 | 0.24 | 4.24 | < .001 |
| **Time Spent Interacting face-to-face (lagged)** | -0.00 | 0.00 | -0.86 | 0.392 |
| **Time Spent in Digitally-Mediated Interactions when Alone (lagged)** | 0.00 | 0.00 | 0.01 | 0.991 |
| **Minutes in Complete Solitude (lagged)** | -0.00 | 0.00 | -0.09 | 0.925 |
| **Extraversion** | 0.14 | 0.08 | 1.71 | 0.090 |
| **Age** | 0.01 | 0.00 | 2.55 | 0.011 |
| **Weekday** | 0.02 | 0.01 | 2.04 | 0.042 |
| **Baseline Loneliness** | -0.05 | 0.05 | -0.92 | 0.357 |
| **Baseline Anxiety** | 0.13 | 0.07 | 1.85 | 0.065 |
| **Baseline Depression** | -0.18 | 0.08 | -2.23 | 0.026 |

### **Momentary dataset – LAPA (Concurrent, Model 1)**

| **Predictor** | ***b*** | ***SE*** | ***t*** | ***p*** |
| --- | --- | --- | --- | --- |
| **Intercept** | -0.03 | 0.53 | -0.05 | 0.961 |
| **Interacting face-to-face** | 0.06 | 0.11 | 0.51 | 0.613 |
| **Extraversion** | 0.15 | 0.31 | 0.49 | 0.632 |
| **Age** | 0.02 | 0.03 | 0.77 | 0.450 |
| **Baseline Loneliness** | -0.02 | 0.17 | -0.16 | 0.872 |
| **Baseline Anxiety** | -0.04 | 0.39 | -0.11 | 0.914 |
| **Baseline Depression** | 0.12 | 0.28 | 0.43 | 0.672 |

### **Momentary dataset - LAPA (Lagged, Model 2)**

| **Predictor** | ***b*** | ***SE*** | ***t*** | ***p*** |
| --- | --- | --- | --- | --- |
| **Intercept** | 0.19 | 0.47 | 0.42 | 0.678 |
| **Digitally-mediated interactions when alone (prior)** | -0.11 | 0.08 | -1.38 | 0.170 |
| **Public solitude (prior)** | -0.00 | 0.08 | -0.04 | 0.969 |
| **Interacting face-to-face (prior)** | -0.18 | 0.09 | -2.06 | 0.041 |
| **Extraversion** | 0.18 | 0.22 | 0.81 | 0.424 |
| **Age** | 0.01 | 0.02 | 0.57 | 0.570 |
| **Baseline Loneliness** | -0.12 | 0.13 | -0.97 | 0.339 |
| **Baseline Anxiety** | 0.23 | 0.26 | 0.95 | 0.352 |
| **Baseline Depression** | 0.02 | 0.16 | 0.13 | 0.895 |

### **Momentary dataset - LAPA (Lagged + prior outcome, Model 3)**

| **Predictor** | ***b*** | ***SE*** | ***t*** | ***p*** |
| --- | --- | --- | --- | --- |
| **Intercept** | 0.37 | 0.39 | 0.96 | 0.347 |
| **Face-to-face (prior)** | -0.04 | 0.10 | -0.43 | 0.667 |
| **Extraversion** | -0.08 | 0.31 | -0.24 | 0.811 |
| **Age** | -0.02 | 0.02 | -0.86 | 0.406 |
| **Baseline Loneliness** | 0.07 | 0.15 | 0.48 | 0.637 |
| **Baseline Anxiety** | 0.30 | 0.42 | 0.72 | 0.485 |
| **Baseline Depression** | 0.40 | 0.29 | 1.37 | 0.194 |
| **Prior LANA** | 0.26 | 0.10 | 2.78 | 0.009 |

## **Table 6 Fixed effects predicting Low Arousal Negative Affect (LANA)**

### **Daily dataset – LANA (Concurrent, Model 1)**

| **Predictor** | ***b*** | ***SE*** | ***t*** | ***p*** |
| --- | --- | --- | --- | --- |
| **Intercept** | 0.45 | 0.17 | 2.60 | 0.009 |
| **Time Spent Interacting face-to-face** | -0.00 | 0.00 | -4.66 | < .001 |
| **Time Spent in Digitally-Mediated Interactions when Alone** | -0.00 | 0.00 | -0.74 | 0.461 |
| **Minutes in Complete Solitude** | -0.00 | 0.00 | -0.79 | 0.429 |
| **Extraversion** | -0.03 | 0.06 | -0.50 | 0.617 |
| **Age** | -0.01 | 0.00 | -3.34 | < .001 |
| **Weekday** | -0.00 | 0.00 | -0.75 | 0.452 |
| **Baseline Loneliness** | -0.05 | 0.04 | -1.37 | 0.172 |
| **Baseline Anxiety** | 0.09 | 0.05 | 1.83 | 0.067 |
| **Baseline Depression** | 0.12 | 0.05 | 2.21 | 0.027 |

### **Daily dataset - LANA (Lagged, Model 2)**

| **Predictor** | ***b*** | ***SE*** | ***t*** | ***p*** |
| --- | --- | --- | --- | --- |
| **Intercept** | 0.32 | 0.17 | 1.83 | 0.068 |
| **Time Spent Interacting face-to-face (lagged)** | -0.00 | 0.00 | -1.25 | 0.210 |
| **Time Spent in Digitally-Mediated Interactions when Alone (lagged)** | 0.00 | 0.00 | 0.59 | 0.553 |
| **Minutes in Complete Solitude (lagged)** | -0.00 | 0.00 | -0.35 | 0.728 |
| **Extraversion** | -0.04 | 0.06 | -0.71 | 0.478 |
| **Age** | -0.01 | 0.00 | -2.89 | 0.004 |
| **Weekday** | 0.00 | 0.01 | 0.18 | 0.860 |
| **Baseline Loneliness** | -0.06 | 0.04 | -1.60 | 0.109 |
| **Baseline Anxiety** | 0.06 | 0.05 | 1.14 | 0.254 |
| **Baseline Depression** | 0.17 | 0.06 | 3.03 | 0.002 |

### **Momentary dataset – LANA (Concurrent, Model 1)**

| **Predictor** | ***b*** | ***SE*** | ***t*** | ***p*** |
| --- | --- | --- | --- | --- |
| **Intercept** | -1.42 | 0.14 | -9.93 | < .001 |
| **Interacting face-to-face** | 0.02 | 0.03 | 0.58 | 0.563 |
| **Extraversion** | -0.04 | 0.09 | -0.42 | 0.676 |
| **Age** | 0.00 | 0.01 | 0.52 | 0.607 |
| **Baseline Loneliness** | 0.05 | 0.05 | 1.11 | 0.278 |
| **Baseline Anxiety** | 0.13 | 0.10 | 1.26 | 0.220 |
| **Baseline Depression** | -0.05 | 0.08 | -0.60 | 0.554 |

### **Momentary dataset - LANA (Lagged, Model 2)**

| **Predictor** | ***b*** | ***SE*** | ***t*** | ***p*** |
| --- | --- | --- | --- | --- |
| **Intercept** | -1.39 | 0.10 | -13.40 | < .001 |
| **Digitally-mediated interactions when alone (prior)** | 0.04 | 0.02 | 1.53 | 0.128 |
| **Public solitude (prior)** | 0.03 | 0.02 | 1.17 | 0.245 |
| **Interacting face-to-face (prior)** | -0.00 | 0.03 | -0.14 | 0.889 |
| **Extraversion** | -0.03 | 0.05 | -0.68 | 0.503 |
| **Age** | 0.00 | 0.01 | 0.10 | 0.921 |
| **Baseline Loneliness** | 0.04 | 0.03 | 1.36 | 0.185 |
| **Baseline Anxiety** | 0.13 | 0.06 | 2.25 | 0.032 |
| **Baseline Depression** | -0.01 | 0.04 | -0.15 | 0.883 |

### **Momentary dataset - LANA (Lagged + prior outcome, Model 3)**

| **Predictor** | ***b*** | ***SE*** | ***t*** | ***p*** |
| --- | --- | --- | --- | --- |
| **Intercept** | -0.90 | 0.14 | -6.60 | < .001 |
| **Interacting face-to-face (prior)** | -0.01 | 0.03 | -0.28 | 0.781 |
| **Extraversion** | -0.06 | 0.06 | -0.98 | 0.346 |
| **Age** | 0.00 | 0.00 | 0.29 | 0.776 |
| **Baseline Loneliness** | -0.02 | 0.03 | -0.76 | 0.458 |
| **Baseline Anxiety** | 0.03 | 0.08 | 0.34 | 0.740 |
| **Baseline Depression** | 0.02 | 0.06 | 0.27 | 0.792 |
| **Prior LANA** | 0.34 | 0.09 | 3.68 | 0.001 |

## **Table 7 Fixed effects predicting High Arousal Positive Affect (HAPA)**

### **Daily dataset - HAPA (Concurrent, Model 1)**

| **Predictor** | ***b*** | ***SE*** | ***t*** | ***p*** |
| --- | --- | --- | --- | --- |
| **Intercept** | 0.01 | 0.17 | 0.06 | .956 |
| **Time Spent Interacting face-to-face** | 0.00 | 0.00 | 7.33 | < .001 |
| **Time Spent in Digitally-Mediated Interactions when Alone** | 0.00 | 0.00 | 2.01 | .045 |
| **Minutes in Complete Solitude** | -0.00 | 0.00 | -1.86 | .063 |
| **Extraversion** | 0.21 | 0.06 | 3.54 | < .001 |
| **Age** | 0.00 | 0.00 | 0.88 | .377 |
| **Weekday** | 0.01 | 0.01 | 1.16 | .248 |
| **Baseline Loneliness** | 0.04 | 0.04 | 1.05 | .293 |
| **Baseline Anxiety** | -0.05 | 0.05 | -0.92 | 0.359 |
| **Baseline Depression** | -0.01 | 0.05 | -0.13 | 0.898 |

### **Daily dataset - HAPA (Lagged, Model 2)**

| **Predictor** | ***b*** | ***SE*** | ***t*** | ***p*** |
| --- | --- | --- | --- | --- |
| **Intercept** | -0.13 | 0.17 | -0.76 | 0.448 |
| **Time Spent Interacting face-to-face (lagged)** | 0.00 | 0.00 | 0.20 | 0.838 |
| **Time Spent in Digitally-Mediated Interactions when Alone (lagged)** | 0.00 | 0.00 | 1.28 | 0.199 |
| **Minutes in Complete Solitude (lagged)** | 0.00 | 0.00 | 0.45 | 0.654 |
| **Extraversion** | 0.22 | 0.06 | 3.67 | < .001 |
| **Age** | 0.00 | 0.00 | 0.56 | 0.573 |
| **Weekday** | 0.01 | 0.01 | 1.88 | 0.060 |
| **Baseline Loneliness** | 0.01 | 0.04 | 0.36 | 0.720 |
| **Baseline Anxiety** | 0.00 | 0.05 | 0.01 | 0.990 |
| **Baseline Depression** | -0.06 | 0.06 | -1.02 | 0.310 |

### **Momentary dataset - HAPA (Concurrent, Model 2)**

| **Predictor** | ***b*** | ***SE*** | ***t*** | ***p*** |
| --- | --- | --- | --- | --- |
| **Intercept** | 1.93 | 0.83 | 2.34 | .023 |
| **Interacting face-to-face** | 0.06 | 0.22 | 0.28 | .779 |
| **Extraversion** | 0.20 | 0.51 | 0.38 | .706 |
| **Age** | -0.08 | 0.04 | -2.19 | .040 |
| **Baseline Loneliness** | -0.53 | 0.28 | -1.92 | .068 |
| **Baseline Anxiety** | -0.52 | 0.63 | -0.83 | .418 |
| **Baseline Depression** | 0.15 | 0.47 | 0.32 | .754 |

### **Momentary dataset - HAPA (Lagged, Model 2)**

| **Predictor** | ***b*** | ***SE*** | ***t*** | ***p*** |
| --- | --- | --- | --- | --- |
| **Intercept** | 2.10 | 0.77 | 2.75 | < .001 |
| **Digitally-mediated interactions when alone (prior)** | 0.07 | 0.17 | 0.42 | .673 |
| **Public solitude (prior)** | 0.14 | 0.17 | 0.83 | .409 |
| **Interacting face-to-face (prior)** | 0.42 | 0.21 | 2.04 | .043 |
| **Extraversion** | -0.19 | 0.37 | -0.50 | .618 |
| **Age** | -0.11 | 0.04 | -2.81 | .008 |
| **Baseline Loneliness** | 0.05 | 0.21 | 0.24 | .812 |
| **Baseline Anxiety** | -0.37 | 0.43 | -0.86 | .396 |
| **Baseline Depression** | 0.24 | 0.26 | 0.91 | .372 |

### **Momentary dataset - HAPA (Lagged, Model 2, moderation analysis)**

| **Predictor** | ***b*** | ***SE*** | ***t*** | ***p*** |
| --- | --- | --- | --- | --- |
| **Intercept** | -0.18 | 0.17 | -1.02 | .306 |
| **Face-to-face time (prior)** | 0.00 | 0.00 | 0.17 | .867 |
| **Virtual time (prior)** | 0.00 | 0.00 | 1.35 | .178 |
| **Minutes in solitude (prior)** | 0.00 | 0.00 | 0.91 | .365 |
| **Extraversion** | 0.43 | 0.09 | 4.54 | < .001 |
| **Age** | 0.00 | 0.00 | 0.59 | .556 |
| **Weekday** | 0.01 | 0.01 | 1.88 | .060 |
| **Baseline loneliness** | 0.01 | 0.04 | 0.31 | .756 |
| **Baseline anxiety** | 0.00 | 0.05 | -0.02 | .986 |
| **Baseline depression** | -0.06 | 0.06 | -1.00 | .319 |
| **Face-to-face × Extraversion** | 0.00 | 0.00 | -0.19 | .851 |
| **Virtual time × Extraversion** | 0.00 | 0.00 | -0.37 | .713 |
| **Minutes in solitude × Extraversion** | -0.00 | 0.00 | -2.91 | .004 |

### **Momentary dataset – HAPA (Lagged + prior outcome, Model 3)**

| **Intercept** | 1.02 | 0.80 | 1.28 | .210 |
| --- | --- | --- | --- | --- |
| **Face-to-face (prior)** | 0.47 | 0.28 | 1.72 | .095 |
| **Extraversion** | 1.20 | 0.60 | 1.99 | .068 |
| **Age** | -0.03 | 0.03 | -1.05 | .311 |
| **Baseline Loneliness** | -0.45 | 0.30 | -1.49 | .161 |
| **Baseline Anxiety** | -0.23 | 0.90 | -0.26 | .801 |
| **Baseline Depression** | -0.68 | 0.65 | -1.04 | .315 |
| **Prior HAPA** | 0.45 | 0.15 | 3.00 | .005 |
| **Intercept** | 1.02 | 0.80 | 1.28 | .210 |
